# CYP2D6*4 Allele Polymorphism Increases the Risk of Parkinson’s Disease: Evidence from Meta-Analysis

**DOI:** 10.1371/journal.pone.0084413

**Published:** 2013-12-20

**Authors:** Yu Lu, Cuiju Mo, Zhiyu Zeng, Siyuan Chen, Yantong Xie, Qiliu Peng, Yu He, Yan Deng, Jian Wang, Li Xie, Jie Zeng, Shan Li, Xue Qin

**Affiliations:** 1 Department of Clinical Laboratory, First Affiliated Hospital of Guangxi Medical University, Nanning, Guangxi, China; 2 Department of Geriatrics, First Affiliated Hospital of Guangxi Medical University, Nanning, Guangxi, China; 3 Guangxi Medical University, Nanning, Guangxi, China; 4 Department of Clinical Laboratory, Liuzhou City People's Hospital, Liuzhou, Guangxi, China; Institut Jacques Monod, France

## Abstract

**Background:**

Many epidemiological studies have been conducted to explore the association between a single CYP2D6 gene polymorphism and Parkinson’s disease (PD) susceptibility. However, the results remain controversial.

**Objectives:**

To clarify the effects of a single *CYP2D6* gene polymorphism on the risk of PD, a meta-analysis of all available studies relating to CYP2D6*4 polymorphism and the risk of PD was conducted.

**Methods:**

A comprehensive literature search of PubMed, EMBASE, and the China National Knowledge Infrastructure (CNKI) up to September 1, 2013 was conducted. Data were extracted by two independent authors and pooled odds ratio (OR) with 95% confidence interval (CI) were calculated. Meta-regression, Galbraith plots, subgroup analysis, sensitivity analysis, and publication bias analysis were also performed.

**Results:**

Twenty-two separate comparisons consisting of 2,629 patients and 3,601 controls were included in our meta-analysis. The pooled analyses showed a significant association between CYP2D6*4G/A polymorphism and PD risk in all of the comparisons (A vs. G allele: OR = 1.28, 95% CI = 1.14–1.43, *P* = 0.001; AA vs. GG: OR = 1.43, 95% CI = 1.06–1.93, *P* = 0.018; AG vs. GG: OR = 1.22, 95% CI = 1.06–1.40, *P* = 0.006; AG+AA vs. GG: OR = 1.26, 95% CI = 1.10–1.44, *P* = 0.001; AA vs. AG+GG: OR = 1.37, 95% CI = 1.02–1.83, *P* = 0.036). In subgroup analysis stratified by ethnicity, significant associations were also demonstrated in Caucasians but not in Asians. No significant association was found in subgroup analysis stratified by age of onset or disease form.

**Conclusions:**

We concluded that the CYP2D6*4G/A polymorphism denotes an increased genetic susceptibility to PD in the overall population, especially in Caucasians. Further large and well-designed studies are needed to confirm this association.

## Introduction

Parkinson’s disease (PD), a progressive neurodegenerative disorder, is clinically characterized by bradykinesia, rigidity, resting tremor, and postural in-stability [1]. The etiology of the disease remains unclear, although a combination of both genetic and environmental factors are believed to cause PD. The environmental etiology of PD was demonstrated following the discovery that a batch of ‘street’ heroin contaminated with 1-methyl-4-phenyl-1,2,5,6-tetrahydropyridine (MPTP) lead to a mimic of idiopathic PD in a group of drug abusers in the early 1980s [2]. On the other hand, several studies have indicated an association between the *CYP2D6* (cytochrome P450, subfamily IID, polypeptide 6; debrisoquine 4-hydroxylase) gene polymorphism and PD in Caucasians [3-5]. Furthermore, it has been reported that organochlorine insecticides are present at a higher concentration in PD tissues, which may partly explain the association between PD and rural living, and, possibly, between PD and polymorphisms within the *CYP2D6* gene [6,7].

*CYP2D6* is a highly polymorphic member of the cytochrome P450 family located on chromosome 22q13 [8]. The major mutation in *CYP2D6* is the G to A substitution at position 1,934 in the junction of intron3/exon4 (CYP2D6*4), also called the *CYP2D6B* or *CYP2D629B* mutant allele [9]. Polymorphism of this gene causes a frame shift in the mRNA and forms a premature stop codon, resulting in a poorer metabolism of debrisoquine 4-hydroxylase [10]. Therefore, it has been postulated that carriers of the mutant *4 allele are more susceptible to neurotoxic damage due to the dysfunctional enzyme and consequently have a higher rate of neuronal loss and reach the disease threshold at an earlier age.

The hypothesis toward poorer metabolism of debrisoquine 4-hydroxylase caused by a defect in *CYP2D6* and risk of PD has been studied by a number of authors; however, results remain controversial. Studies by Armstrong et al. [9] and Smith et al. [11] reported that the frequency of mutations in CYP2D6*4 confer a two-fold increase in odds ratio (OR) for PD patients compared to healthy control individuals. On the other hand, no significant difference in mutation frequency was found in studies conducted by Plante-Bordeneuve et al. [12] and Atkinson et al. [13]. Determination of this polymorphism, which accounts for the highest frequency of *CYP2D6* mutant alleles (21%) [14], could help identify whether *CYP2D6* polymorphisms denote a susceptibility to developing PD. We therefore performed a systematic review and meta-analysis to quantitatively assess the association between CYP2D6*4 allele polymorphism and PD risk.

## Methods

### Search strategy

A comprehensive literature search of PubMed, EMBASE, and the China National Knowledge Infrastructure (CNKI) covering all articles published up to September 1, 2013 was conducted using the search strategy based on combinations of the keywords “*cytochrome P450 CYP2D6* OR *CYP2D6*” and “Parkinson OR Parkinson’s *disease*” and “*polymorphism*, *mutation* OR *variant*”. No language restrictions were applied. The reference lists of relevant articles were checked manually to find additional eligible studies and no attempts were made to identify unpublished studies. 

### Selection criteria

This study was performed according to the Meta-Analysis of Observational Studies in Epidemiology (MOOSE) guidelines for reporting [15]. Inclusion criteria were defined as follows: i) studies evaluating the association between CYP2D6*4 allele polymorphism and PD; ii) case-control studies; and iii) studies with sufficient data available to estimate the OR with their 95% confidence intervals (95% CIs). Exclusion criteria included: i) articles without sufficient information for data extraction and ii) articles that only assessed the association between *CYP2D6* and PD in phenotype or other alleles. If dual or multiple studies were reported by the same institution or authors, either the one of higher quality or the most recent study was included in the analysis.

### Data extraction

Two investigators (Xue Qin and Yu Lu) independently extracted data from the studies included. The following variables were extracted: first author’s name, year of publication, country, ethnicity, genotyping method, number of cases and controls, source of controls, genotype distribution in cases and controls and *P* value for control population in Hardy-Weinberg equilibrium (HWE), and the onset age of PD patients as well as the form of the disease (sporadic or familial). If different results were generated, discrepancies were discussed to reach consensus. Considering the subgroup of ethnicity, subjects were grouped into the main racial group of the study population according to their geographical origin or ancestry [16].

### Quality assessment

To evaluate the quality of the included studies, two investigators (Cuiju Mo and Zhiyu Zeng) assessed them independently based on a set of predetermined criteria which was initially proposed by Thakkinstian et al. [17] and has been in a previous meta-analysis [18] (Table S2). The predetermined criteria were scored from 0 (lowest) to 15 (highest) to classify the studies into high or low quality studies. Studies with scores of ≥10 or <10 were considered as high- and low-quality studies, respectively. Any disagreement was resolved by consensus between the two authors. A third reviewer (Shan Li) was invited to the discussion if disagreement prevailed.

### Statistical analysis

For the included studies, summary ORs and corresponding 95% CIs were used as a measure to assess the association of CYP2D6*4 allele polymorphism and PD risk. The following contrasts were evaluated: i) a comparison of variant alleles with a wild allele (A vs. G allele); ii) a comparison of each homozygote with the other combined with a heterozygote (AG+AA vs. GG; AA vs. GG+AG); and iii) a comparison of a variant homozygote with a heterozygote and a wild homozygote (AA vs. GG; AG vs. GG). Subgroup analyses were also conducted to evaluate the effect of CYP2D6*4 allele polymorphism on the susceptibility to PD in different populations (Asian and Caucasian); different onset age of PD patients (grouped as early onset age (E): <51 years old, late onset age (L): >51 years old; and not available (NA)); and different forms of PD (sporadic (S)/familial (F)/not reported (NR)). The Q test and I^2^ statistics for heterogeneity were carried out for each combined analysis where *P*_Q_ <0.10 or I^2^ >50% indicated significant heterogeneity. If significant heterogeneity existed, a random-effects model (the DerSimonian and Laird method) was selected to pool the data; otherwise, a fixed-effects model (the Mantel-Haenszel method) was used. When heterogeneity was detected, we performed logistic meta-regression to explore the sources of heterogeneity among studies. The following characteristics were included as covariates in the analysis: genotyping methods (PCR-RFLP/not PCR-RFLP), ethnicity (Caucasian/Asian), quality score (high/low), source of controls (hospital-based/not hospital-based), onset age of PD (E/L/NA), and form of the disease (S/F/NR). Galbraith plot analysis was performed for further exploration of the heterogeneity.

To identify possible influential studies, sensitivity analysis was also performed by sequential omission of individual studies, excluding those without definite diagnostic criteria as well as those in which genotype frequencies in control populations exhibited significant deviation from the HWE, given that the deviation may denote bias. HWE in the control group population was tested by using a goodness-of-fit Chisquare test. For each polymorphism, publication bias was evaluated using a funnel plot and Egger’s regression asymmetry test (*P* <0.05 was considered a significant publication bias). All data were analyzed using STATA software version 11.0 (Stata Corp, College Station, TX, USA) and all *P* values were two-sided.

## Results

### Study characteristics

**Figure 1:**
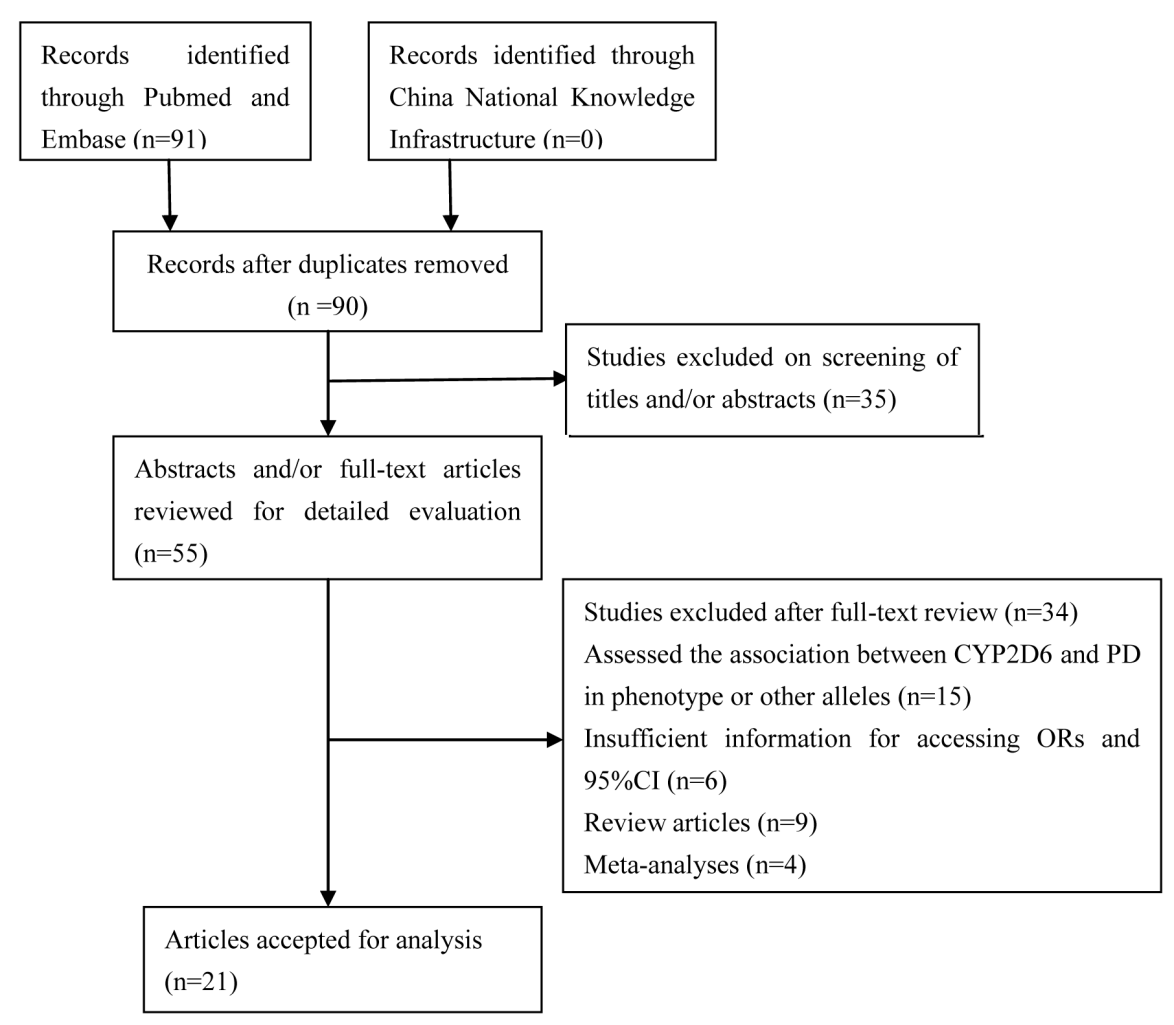
Flow diagram for the selection of articles for inclusion in the meta-analysis.

A total of 91 studies were identified; 35 studies that did not focus on *CYP2D6* were excluded after reviewing the title. After a careful abstract and/or full-text review, a further 35 studies were excluded: 15 assessed the association between *CYP2D6* and PD in phenotype or other alleles, 6 without sufficient information to access ORs and 95% CI, 13 were review articles or meta-analyses, and 1 had duplicated data. Thus, 21 relevant studies that focused on CYP2D6*4 allele polymorphism and PD risk met the inclusion criteria for the meta-analysis [9,11-13,19-35]. The retrieval process is illustrated in Figure 1. Among the eligible studies, one contained data on two different forms of the disease (sporadic and familial) [28], and the results were therefore treated independently. Consequently, 22 separate comparisons consisting of 2,629 patients and 3,601 controls were finally included in the meta-analysis. The main characteristics of the studies are presented in Table 1. Of all the eligible studies, 15 were conducted in Caucasians and 6 in Asians; 4 studied PD patients with early onset age and 5 with late onset age; and 13 were conducted in sporadic patients and 6 in familial patients. The genotype distributions of the controls in 4 studies were not consistent with HWE. Sixteen studies had quality scores of >10 and the rest of <10. Details are presented in Table 2.

**Table 1:** Characteristics of studies included in this meta-analysis.

Author, Year	country	Ethnicity	case/control	Genotyping methods	Form of PD patients(S/F/NR)	PD on-set age(E/L/NA)	Source of control	PI	HWE(P value)	QS
Smith CA,1992	UK	Caucasian	193/720	PCR	S	NA	H-B	CYP2D6*4 G>A	0.106	12
Armstrong M,1992	UK	Caucasian	53/72	PCR-RFLP	NR	NA	H-B	CYP2D6-B	0.174	10
Kurth MC,1993	USA	Caucasian	50/110	PCR-RFLP	F	L	H-B	CYP2D6-29B	0.178	10
Plante-Bordeneuve,1994	British and Irish	Caucasian	45/69	PCR	F	NA	H-B	CYP2D6-B	0.215	10
Akhmedova SN,1995	Russia	Caucasian	80/70	PCR-RFLP	S	NA	F-B	CYP2D6-29B	0.886	9
Agundez JA,1995	Spain	Caucasian	123/150	PCR-RFLP	NR	33/123 E: 39.4±7.2y 90/123 L: 65.9±8.0y	P-B	CYP2D6-B	0.552	11
Diederich N,1996	Germany	Caucasian	80/106	PCR	S	32/80 E: 39.8±5.1y 48/80 L: 63.2±7.7y	Mix	CYP2D6-B	0.014	7
Gasser T,1996	diverse	Caucasian	178/73	PCR	115/178 S 63/178 F	NA	H-B	CYP2D6-B	0.901	10
Chen X,1996	guam	Oriental population	28/212	PCR	S	NA	H-B	CYP2D6-B	0.033	7
Bordet R,1996	France	Caucasian	105/105	PCR-RFLP	S	NA	H-B	CYP2D6-B	0.065	10
Pang CP,1997	China	Asian	200/227	PCR	S	NA	H-B	CYP2D6*4 G>A	0.925	11
Chen DK,1998	China	Asian	215/313	PCR	S	NA	H-B	CYP2D6*4 G>A	0.936	11
Nicholl,1999	UK	Caucasian	176/175	PCR-RFLP	S	NA	H-B	CYP2D6*4 G>A	0.066	10
Nicholl,1999	UK	Caucasian	30/30	PCR-RFLP	F	NA	H-B	CYP2D6*4 G>A	0.110	9
Atkinson A,1999	UK	Caucasian	33/75	PCR-RFLP	S	NA	H-B	CYP2D6*4 G>A	0.019	8
Chida M,1999	Japan	Asian	38/270	PCR	S	NA	H-B	CYP2D6*4 G>A	0.862	10
Joost O,1999	USA	Caucasian	109/110	PCR	NR	NA	H-B	CYP2D6*4 G>A	0.618	10
Štefanovć M,2000	Croatia	Caucasian	41/145	LR-PCR	NR	NA	H-B	CYP2D6*4 G>A	0.495	10
PayamiH,2001	USA	Caucasian	576/247	PCR	360/576 S 162/576 F	145/576E: 41.1±7.8y 431/576 L: 63.1±7.7y	H-B	CYP2D6*4 G>A	0.004	9
Woo SI,2001	Korea	Asian	93/122	PCR	NR	NA	H-B	CYP2D6*4 G>A	0.945	10
Durić G,2007	Russia	Caucasian	106/75	PCR–RFLP	96/106 S 10/106 F	55/106 E: 39.5±3.8y 41/106 L: 58.1±6.2y	H-B	CYP2D6*4 G>A	0.281	11
Singh M,2010	India	Asian	77/125	PCR–RFLP	S	NA	H-B	CYP2D6*4 G>A	0.059	11

PI, Polymorphism(s) investigated; PCR–RFLP, Polymerase chain reaction-restriction fragment length polymorphism; RT–PCR, Real time-polymerase chain reaction; LR-PCR, long-range-PCR; NR: Not report; NA, Not available; PB, Population–based; HB, Hospital–based; HWE, Hardy–Weinberg equilibrium in control population; QS: quality score

**Table 2:** Meta-analysis and heterogeneity test of the CYP2D6 gene polymorphisms on PD risk.

Comparisons	No. of study	No. of patient	A vs. G		AA vs. GG		AG vs. GG		AG + AA vs. GG		AA vs. AG + GG	
			OR(95%CI)	I^2^(%)	OR(95%CI)	I^2^(%)	OR(95%CI)	I^2^(%)	OR(95%CI)	I^2^(%)	OR(95%CI)	I^2^(%)
overall	22	2629	1.28(1.14-1.43)	38.7	1.43(1.06-1.93)	0.0	1.22(1.06-1.40)	29.8	1.26(1.10-1.44)	23.1	1.37(1.02-1.83)	0.0
subgroup												
Sporadic/Familial PD(S/F/NR)												
S	13	1932	1.15(1.00-1.32)	17.6	1.40(1.00-1.96)	11.0	1.06(0.89-1.25)	13.4	1.11(0.95-1.30)	0.0	1.37(0.98-1.92)	21.0
F	6	278	1.40(1.04-1.88)	74.1	1.13(0.56-2.29)	26.4	1.50(1.04-2.16)	58.1	1.44(1.02-2.03)	64.6	1.33(1.00-1.78)	2.7
NA	5	419	1.65(1.24-2.20)	23.4	1.80(0.71-4.59)	9.5	1.62(1.17-2.26)	0.0	1.65(1.19-2.27)	0.0	1.61(0.64-4.08)	3.9
Early/Late-onset PD(E/L/NA)												
E	4	265	0.84(0.62-1.14)	85.1	0.60(0.28-1.27)	0.0	0.97(0.67-1.41)	80.8	0.90(0.63-1.28)	81.1	0.66(0.32-1.50)	0.0
L	5	660	1.15(0.93-1.42)	74.4	0.87(0.52-1.47)	37.8	1.28(0.98-1.66)	34.6	1.20(0.94-1.54)	55.2	0.80(0.48-1.33)	5.9
NA	16	1694	1.32(1.14-1.53)	33.9	1.67(1.14-2.45)	0.0	1.23(1.03-1.46)	31.9	1.28(1.09-1.52)	24.5	1.61(1.10-2.34)	0.0
Ethnicity												
Asian	6	651	1.26(0.78-2.03)	0.0	1.08(0.21-5.47)	0.0	1.31(0.77-2.23)	0.0	1.27(0.76-2.13)	0.0	1.05(0.21-5.28)	0.0
Caucasian	15	1978	1.28(1.13-1.44)	50.2	1.45(1.07-1.96)	0.3	1.21(1.05-1.40)	39.4	1.26(1.09-1.44)	33.4	1.38(1.02-1.86)	1.4

OR: odds ratio; CI: confidence intervals; S/F/NR: Sporadic/Familial/Not Report; E/L/NA: Early/Late/Not Available.

### Meta-analysis results

The pooled analysis suggested that the CYP2D6*4 allele polymorphism was significantly associated with an increased risk of PD in all genetic models in the overall population: i) A vs. G allele (OR = 1.28, 95% CI = 1.14–1.43, *P* = 0.001); ii) AA vs. GG (OR = 1.43, 95% CI = 1.06–1.93, *P* = 0.018); iii) AG vs. GG (OR = 1.22, 95% CI = 1.06–1.40, *P* = 0.006); iv) AG+AA vs. GG (OR = 1.26, 95% CI = 1.10–1.44, *P* = 0.001); and v) AA vs. AG+GG (OR = 1.37, 95% CI = 1.02–1.83, *P* = 0.036). In the subgroup analysis stratified by ethnicity, the results also showed a significant contribution of the CYP2D6*4 allele polymorphism to PD development in the Caucasian population in all of the comparisons of the A vs. G allele (OR = 1.28, 95% CI = 1.13–1.44, *P* = 0.001); AA vs. GG (OR = 1.45, 95% CI = 1.07–1.96, *P* = 0.017); AG vs. GG (OR = 1.21, 95% CI = 1.05–1.40, *P* = 0.009); AG+AA vs. GG (OR = 1.26, 95% CI = 1.09–1.44, *P* = 0.001); and AA vs. AG+GG (OR = 1.37, 95% CI = 1.02–1.86, *P* = 0.034). When stratified by form of the disease, a significantly increased risk was found in all forms of the disease, indicating that susceptibility to PD due to CYP2D6*4 allele polymorphism may be independent from the form of PD onset. Similarly, a significantly increased risk was found only in the “Not available” group when stratified by PD onset age, suggesting that CYP2D6*4 allele mutations are independent from onset age. The details are presented in Table 1.

### Heterogeneity analysis

**Figure 2:**
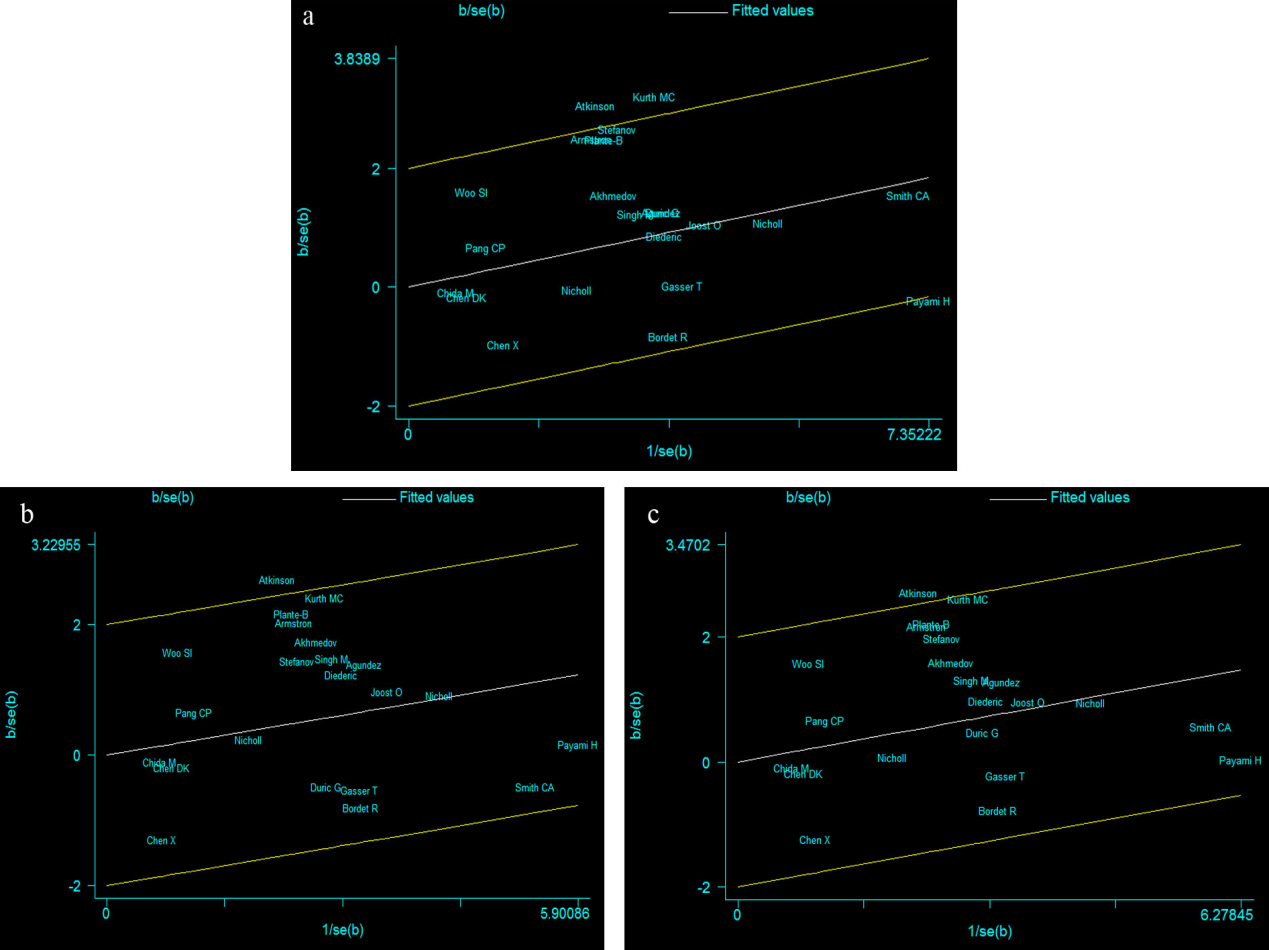
Galbraith plots of CYP2D6*4 allele polymorphisms and PD risk in different contrast models. a The studies of Kurth MC et al. and Atkinson A et al. were outliers in the contrast A vs. G. b The studiy of Atkinson A et al. was the outlier in the contrast AG vs. GG. c The studies of Atkinson A et al. was the outlier in the contrast AG+AA vs. GG.

For the A vs. G comparison model, the I^2^ value of heterogeneity was lower than 50% and the P_Q_ value was lower than 0.10 (I^2^=37.8%, *P*_Q_= 0.034), indicating a statistically significant heterogeneity among studies. To explore the sources of heterogeneity, we performed meta-regression analyses. The results showed that none of the factors mentioned above were effect modifiers: genotyping methods (*P* = 0.873), ethnicity (*P* = 0.853), quality score (*P* = 0.292), source of controls (*P* = 0.166), onset age of PD (*P* = 0.423), and form of the disease (*P* = 0.948). To further investigate the heterogeneity, Galbraith plots analysis was performed to identify the outliers that might contribute to the heterogeneity. Our results showed that the studies by Kurth et al. [19] and Atkinson et al. [13] were the outliers and main contributors to heterogeneity in this comparison model (Figure 2a). A forest plot omitting the outlier studies was conducted, the significance of the OR was not altered (OR = 1.21, 95% CI = 1.08–1.37, *P* = 0.001) and was without evidence of heterogeneity (I^2^ = 18.0%, P_Q_ = 0.230) in the overall populations as well as in subgroup analysis of the Caucasian population (OR = 1.21, 95% CI = 1.07–1.37, *P* = 0.002, I^2^ = 31.5%, *P*_Q_ = 0.124). However, results remained the same in the other subgroup analyses. 

For the remaining comparison models, there was no statistically significant heterogeneity in the overall population. However, subgroup analysis by onset age and form of the disease indicated significant heterogeneity in the AG vs. GG and AG+AA vs. GG models. When the Galbraith plot was analyzed, the study by Atkinson et al. [13] was identified as the main contributor to heterogeneity (Figure 2b,c); however, after exclusion of the study, heterogeneity still existed among studies in the two genetic comparison models mentioned above (data not show).

### Sensitivity analysis

As the genotype frequencies of the control group in 4 studies deviated significantly from HWE [13,23,25,32] (Table 2), the influence of each study involved in the meta-analysis of the pooled ORs was examined by rerunning the meta-analysis with one study excluded each time. Results showed that the corresponding pooled ORs were not significantly altered (data not shown), indicating that our results were statistically robust.

### Publication bias

**Figure 3:**
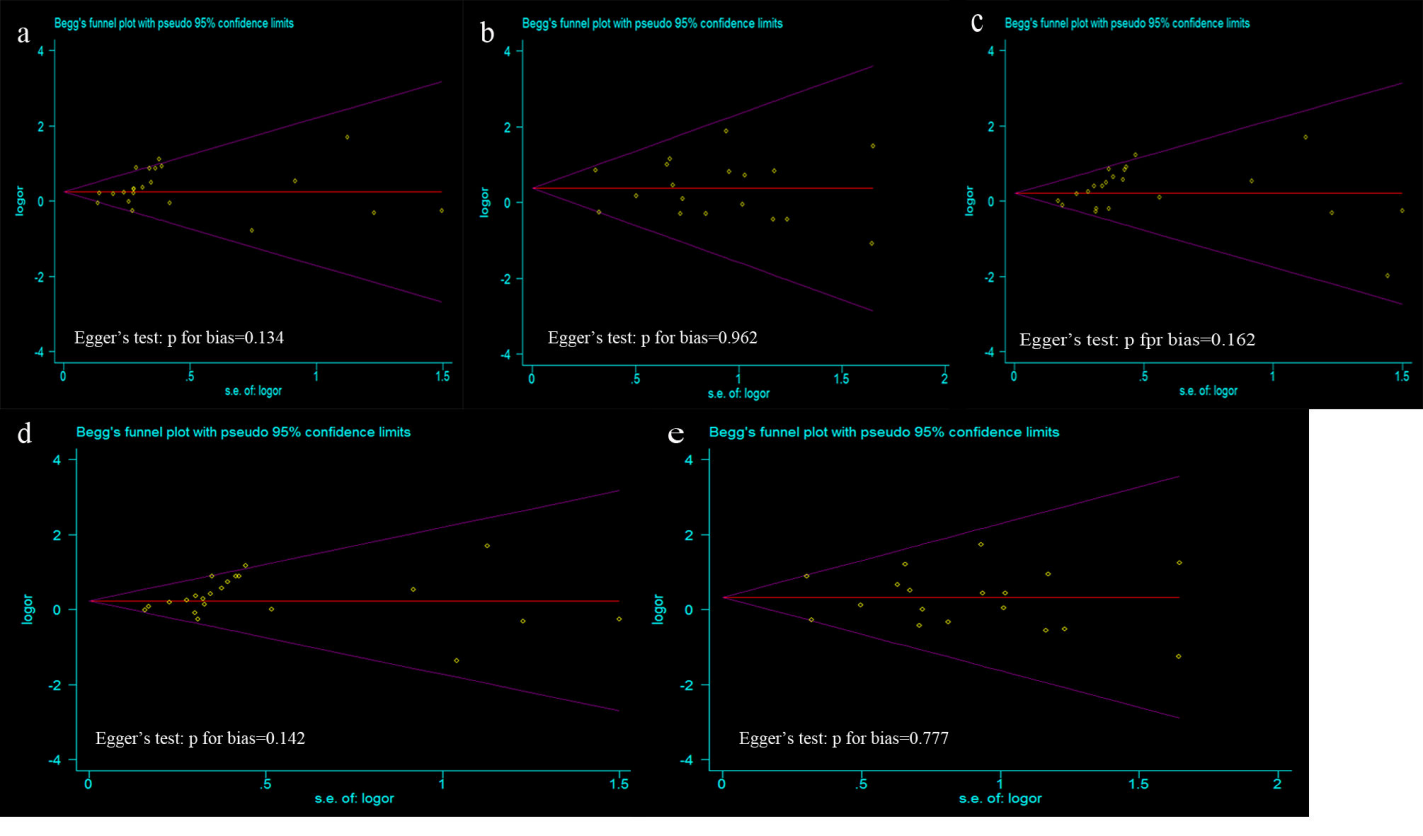
Funnel plot analysis and Egger’s test to detect publication bias in different contrast models. Each point represents a separate study for the indicated association. a Funnel plot and Egger’s test for contrast A vs. G; b Funnel plot and Egger’s test for contrast AA vs. GG; c Funnel plot and Egger’s test for contrast AG vs. GG; d Funnel plot and Egger’s test for contrast AG+AA vs. GG; e Funnel plot and Egger’s test for contrast AA vs. GG+AG.

Begg’s funnel plot and Egger’s test were performed to assess the publication bias of articles in all comparison models. The shape of the funnel plots were symmetrical, suggesting that there was no evidence of publication bias among the studies. Egger’s test was also used to statistically assess the funnel plot symmetry. The results still did not suggest any evidence of publication bias in all comparison models (Figure 3).

## Discussion

*CYP2D6* is a member of the cytochrome P450 gene family, which plays an important role in the metabolism of many substances [8]. It has been reported that *CYP2D6* is expressed constitutively in human liver and metabolizes a variety of therapeutic drugs, including antiarrhythmics, antidepressants, and neuroleptics [36]. Evidence that *CYP2D6* can metabolize environmental toxins such as the neurotoxins 1,2,3,4-tetrahydroisoquinoline and MPTP has also been found [37,38]. A common variation in the *CYP2D6* gene is a G to A shift in exon4, which results in substitution of Glu for Gly [39], and is associated with reduced *CYP2D6* activity. Decreased *CYP2D6* activity may lead to an alteration of susceptibility to neurotoxic damage and a higher rate of neuronal loss, thus making carriers more susceptible to PD; this hypothesis was confirmed by the present meta-analysis.

In our study, a significantly increased risk of PD was associated with the CYP2D6*4A allele in the overall population and in Caucasians in particular, suggesting that individuals with the mutated CYP2D6*4 genotype had higher risk of PD compared to those with wild type, especially among the Caucasian population. However, no association was detected among the Asian population. Indeed, it is common for the same polymorphism to play different roles in disease susceptibility among different ethnic populations. This may be principally explained by the *CYP2D6* genetic background difference between such populations [40]; using allele-specific PCR, approximately 100 Caucasian subjects were screened for the presence of CYP2D6*4G/A mutation, but in the original population studied, the mutation was only detected in one individual [39]. Further, multiple environmental factors, such as diet, residence in rural areas, and exposure to pesticides, that may be involved in the mechanism linking *CYP2D6* genotype and the risk of developing PD vary between ethnic groups [41]. Finally, considering the limited number of Asian-patient studies included in the meta-analysis (Asian patients = 651, Caucasian patients = 1,978), the differences may have arisen by chance. Hence, our results should be interpreted with caution.

In the subgroup analysis based on form of the disease, a significantly increased risk was found for all forms of the disease (familial, sporadic, not reported), suggesting that the presence of CYP2D6*4 polymorphism leading to poor debrisoquine metabolism is associated with an increased risk for PD regardless of the patient’s family history. However, there is considerable variation in the frequencies of mutant *CYP2D6* alleles in familial and sporadic PD of different populations [11,22,42,43]. Nevertheless, the majority of studies included in our subgroup analysis did not reveal any significant association between *CYP2D6* mutations and familial PD [12,19,22,32,34]. Such discrepancy is probably not the result of population bias but likely the involvement of a multitude of genetic and environmental factors because there is no evidence of clinical or histological difference in PD in different populations [22].

In a further subgroup analysis restricted to the onset age of PD patients, no significant increased risk was found in patients with early or late onset age, suggesting that the early-onset PD patients share the same genetic background as the late-onset PD patients and there might be not any different pathogenetic mechanisms involved in onset age of PD patients. It has been reported that the frequency of *4 allele is significantly elevated in late-onset as compared with early-onset PD, but similar trend was also found in non-PD populations [32]. Therefore, the difference in *4 allele frequency between early- and late-onset PD observed by some studies was probably due to an age effect but does not necessarily signify an association with disease.

This meta-analysis should be considered with its limitations. First, the *CYP2D6* phenotypes were not studied. Genetic polymorphism of *CYP2D6* is inherited as an autosomal recessive trait, with more than 60 alleles known thus far (http://www.cypalleles.ki.se/). These alleles, occurring in various combinations or along with other alleles, result in three phenotypes: extensive metabolizer, intermediate metabolizer, and poor metabolizer [44,45]. Determination of these phenotypes, which contain all genetic polymorphisms of *CYP2D6*, can comprehensively and directly access the relationship between *CYP2D6* polymorphism and PD risk. Second, the overall outcomes of our study were based on individual unadjusted ORs, but a more precise evaluation should be adjusted by potentially suspected factors such as age, gender, and environmental factors. Such a problem was observed in a meta-analysis conducted by Rostami et al. [46], where a marginally higher poor metabolizer phenotype frequency in patients was found, but the ORs dropped to 1 when studies that did not match patients and control subjects for age were excluded. Some studies included in our meta-analysis also did not use subjects matched for age or environmental factors; the results would hence underestimate the association of OR with genotype. Third, the controls were not uniformly defined. Though most of the controls were selected mainly from healthy populations, there are some were unaffected relatives with a positive family history or benign disease such as chronic bronchitis was included [22,24]. Hence, as these studies may have included individuals with different risks of developing PD in their control groups, a non-differential misclassification bias was possible. Fourth, the number of studies on CYP2D6*4 polymorphism in the Asian population included in this study was relatively small, which may lead to low statistical power. Finally, publication bias was possible as no attempts were made to identify unpublished studies, though results of our Begg’s funnel plot and Egger’s test revealed no evidence of publication bias in all comparison models.

In conclusion, the present meta-analysis clearly indicates that the CYP2D6*4G/A polymorphism denotes an increased genetic susceptibility to PD in the overall population, but particularly in Caucasians. However, larger sample studies with a more rigorous design and well-matched controls are required to overcome the above-mentioned limitations, especially investigations taking the other allele polymorphisms of *CYP2D6* as well as phenotype status into consideration.

## Supporting Information

Table S1 **Check list.**
(DOC)

Table S2 **Scale for quality assessment.**
(DOC)
