# Supplementary material for: CYP2D6*4 Allele Polymorphism Increases the Risk of Parkinson’s Disease: Evidence from Meta-Analysis
Source: PLoS One. 2013 Dec 20;8(12):e84413. doi: 10.1371/journal.pone.0084413 (PMC3869836; doi:10.1371/journal.pone.0084413)
Supplement: Table S2 — Scale for quality assessment. (DOC) [file pone.0084413.s002.doc]

Table S2 Scale for quality assessment[[1](#_ENREF_1)]

| Criteria | Score |
| --- | --- |
| Representativeness o f cases |  |
| Selected from population or disease registry | 3 |
| Selected from hospital | 2 |
| Selected from pathology archives, but without description | 1 |
| Not described | 0 |
| Credibility of controls |  |
| Population- based | 3 |
| Blood donors or volunteers | 2 |
| Hospital-based | 1 |
| Not described | 0 |
| Specimens of cases determining genotypes |  |
| White blood cells or normal tissues | 3 |
| histopathology or exfoliated cells of tissue | 0 |
| Hardy-Weinberg equilibrium in controls |  |
| Hardy-Weinberg equilibrium | 3 |
| Hardy-Weinberg disequilibrium | 0 |
| Total sample size |  |
| 》1000 | 3 |
| 》400 but <1000 | 2 |
| 》200 but <400 | 1 |
| <200 | 0 |

1. Qin X, Peng Q, Chen Z, Deng Y, Huang S, Xu J et al. The association between MTHFR gene polymorphisms and hepatocellular carcinoma risk: a meta-analysis. PLoS One. 2013;8(2):e56070. doi:10.1371/journal.pone.0056070.
